# Internal Iliac Aneurysm Causing Hydroureteronephrosis

**DOI:** 10.1155/2020/8857729

**Published:** 2020-11-12

**Authors:** Tawfeeq Sangey, Sibtain Moledina

**Affiliations:** ^1^Regency Medical Centre, Tanzania; ^2^Shree Hindu Mandal Hospital, Tanzania

## Abstract

A 63-year-old presented with right lumbar pain and increased frequency of urination. Imaging revealed right internal iliac artery aneurysm causing hydroureteronephrosis and compressing the urinary bladder.

## 1. Case Presentation

**Figure 1:**
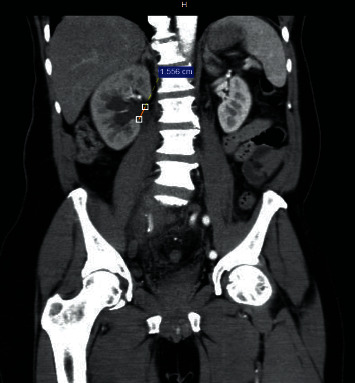
CT-IVU showing the right sided hydroureteronephrosis

**Figure 2:**
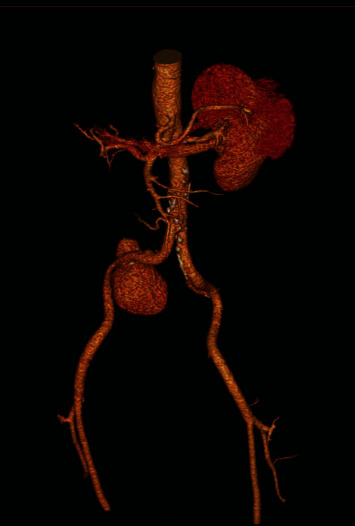
Multiplanar reformatted images show right internal iliac artery aneurysm just past the iliac bifurcation.

**Figure 3:**
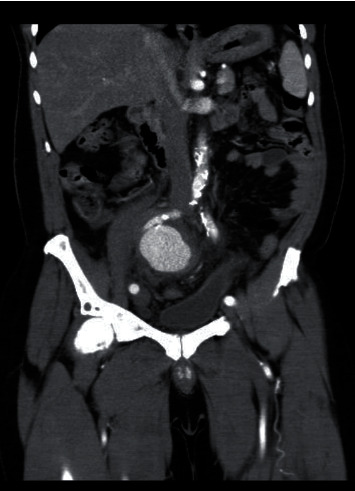
Contrast-enhanced CT scan of the abdomen and pelvis, with coronal images showing a large contrast-filled structure in keeping with iliac artery aneurysm compressing and pushing the urinary bladder laterally.

A 63-year-old male presented at the urology clinic with complaints of right lumbar pain and increased frequency of urination. He carried a recent ultrasound exam showing a grade 2 right ureterohydronephrosis. A repeat ultrasound scan was performed. B-mode ultrasound demonstrated a large saccular type of lesion with a thick pulsating wall connected by feeding artery arising from the right iliac artery and intramural thrombus formation. Colour mode ultrasound confirmed the turbulent blood flow within the aneurysm. The prostate was mildly enlarged at 34 grams associated with a moderate residual volume of 61 cc. CT-IVU confirmed the right ureterohydronephrosis with an anteroposterior diameter of the renal pelvis of 15 mm (Figure 1). There was no evidence of renal or ureteric stones. A right internal iliac artery aneurysm was noted at the level of L4/L5 vertebra bodies measuring 6.7 × 6.0 × 6.05 cm (AP × trans × Sag) (Figure 2), and the right ureter was moderately dilated to the level of the right internal iliac aneurysm (Figure 3), which was possibly the cause of the obstruction.

The urinary bladder was superiorly compressed and laterally pushed on the left iliac fossa region by the aneurysm.

The patient was planned for surgical intervention and transferred to a specialized facility for surgery. Unfortunately, the patient succumbed to the illness while waiting for surgery due to rupture of the aneurysm,

## 2. Discussion

Aneurysms of the iliac arteries are found considerably less often. Most of the internal iliac artery (IIA) aneurysms occur in association with other intra-abdominal aneurysms (abdominal aorta, common, infrarenal, and iliac arteries) making up part of the polyaneurysm disease [1].

The incidence of iliac artery in conjunction with aneurysms of the abdominal aorta is approximately 10% but isolated iliac aneurysms are rare and occur in only 2% [2].

The majority of patients are elderly aged 65–75 years and commonly seen in males with a ratio 6 times more than in females [3].

IIA aneurysms are usually asymptomatic, due to the deep location of the internal iliac artery and can occur in the retroperitoneal or intraperitoneal spaces, compressing the rectum, ureter, or bladder triggering urological, gastroenterological, and neurological symptoms [4–6].

Compression of the ureter and bladder triggering the urinary symptoms has also been reported [7, 8].

Due to their deep location in the pelvis and the fact that they often are asymptomatic, diagnosis is often delayed until the aneurysm is of a significant size producing symptoms or coincidentally found by radiological imaging for other reasons.

The incidence of rupture is high and may be up to 38% at initial presentation. This has been reported to carry a 58% mortality rate [9]. The mean diameter of the aneurysm at the time of rupture is almost 7 cm delaying operative treatment until a diameter of 4 cm may be safe.

Ultrasound is useful in an initial investigation as it depicts the ureterohydronephrosis and other urinary track complications. With colour-flow Doppler, the blood flow within an aneurysm can be confirmed [10].

Helical computed angiotomography is the gold standard, showing the site, size, tortuosity, path, relationship with adjacent organs, signs of rupture, and retroperitoneal hemorrhage [4, 6, 11].

## Data Availability

Data supporting the information in this case report are in the patient files which are the property of the hospital where he was seen.
